# Supplementary material for: A Novel Four-Gene Score to Predict Pathologically Complete (R0) Resection and Survival in Pancreatic Cancer
Source: Cancers (Basel). 2020 Dec 4;12(12):3635. doi: 10.3390/cancers12123635 (PMC7761977; doi:10.3390/cancers12123635)
Supplement: Supplementary file 1 [file cancers-12-03635-s001.pdf]

*SUPPLEMENTAL MATERIALS*

# Oshi et al. “A novel 4-gene score to predict pathologically complete (R0) resection and survival in pancreatic cancer”

1. Table S1: Comparison of clinical and pathological features between low and high 4-gene score with pancreatic cancer in the TCGA cohort.
2. Table S2: Comparison of pathological features between low and high 4-gene score with pancreatic cancer in the GSE62452 cohort.
3. Table S3: Comparison of pathological feature between low and high 4-gene score with pancreatic cancer in the GSE57495 cohort.
4. Table S4: Pancreatic cancer cell lines
5. Figure S1: Gene set enrichment analysis (GSEA) of high 4-gene score pancreatic cancer in the TCGA, GSE62452, and GSE57495 cohorts.

**Table S1.** Comparison of clinical and pathological features between low and high 4-gene score with pancreatic cancer in the TCGA cohort. .

| **TCGA (*n* = 176)** | **Low (88)** | **High (88)** | ***p*-value** |
|---|---|---|---|
| **Age** | | | 0.602 |
| Median | 66 | 65 | |
| IQR | 58-72 | 57-73 | |
| **Race** | | | 0.450 |
| White | 80 | 75 | |
| Black | 2 | 4 | |
| Asian | 4 | 7 | |
| Unknown | 2 | 2 | |
| Histological type | | | 0.112 |
| PDAC* | 68 | 77 | |
| Other | 20 | 11 | |
| Tumor site | | | 0.68 |
| Body/Tail | 15 | 13 | |
| Head | 66 | 71 | |
| Unknown | 7 | 4 | |
| **T-category** | | | 0.017 |
| T1 | 3 | 4 | |
| T2 | 18 | 5 | |
| T3 | 64 | 77 | |
| T4 | 1 | 2 | |
| Unknown | 2 | 0 | |
| **N-category** | | | 0.178 |
| N- | 28 | 21 | |
| N+ | 55 | 67 | |
| Unknown | 5 | 0 | |
| **M-category** | | | 1.00 |
| M- | 36 | 42 | |
| M+ | 2 | 2 | |
| Unknown | 50 | 44 | |

*PDAC: pancreatic ductal adenocarcinoma.

**Table S2: Comparison of pathological features between low and high 4-gene score with pancreatic cancer in the GSE62452 cohort.** .

| GSE62452 (*n* = 69) | Low (34) | High (35) | *p*-value |
|---|---|---|---|
| **Grade** | | | 0.024 |
| 1 | 2 | 0 | |
| 2 | 21 | 14 | |
| 3 | 10 | 20 | |
| unknown | 1 | 1 | |
| **Stage** | | | 0.706 |
| I | 3 | 1 | |
| II | 23 | 23 | |
| III | 5 | 8 | |
| IV | 3 | 3 | |
| Unknown | 0 | 0 | |

**Table S3.** Comparison of pathological feature between low and high 4-gene score with pancreatic cancer in the GSE57495 cohort. .

| GS57495 (*n* = 63) | Low (31) | High (32) | *p*-value |
|---|---|---|---|
| **Stage** | | | 0.889 |
| I | 0 | 2 | |
| IB | 7 | 5 | |
| IIA | 8 | 9 | |
| IIB | 26 | 17 | |
| Unknown | 0 | 0 | |

**Table S4.** Pancreatic cancer cell lines.

| Primary | Metastasis |
|---|---|
| BXPC3 | ASPC1 |
| CAPAN2 | CAPAN1 |
| HPAC | CFPAC1 |
| KP2 | HPAFII |
| MIAPACA2 | HS766T |
| PANC0203 | HUPT3 |
| PANC0327 | HUPT4 |
| PANC0403 | KP3 |
| PANC0504 | KP4 |
| PANC0813 | L33 |
| PANC1005 | PATU8988S |
| PATU8902 | PATU8988T |
| PK45H | PK1 |
| PSN1 | PK59 |
| QGP1 | SNU410 |
| SW1990 | SU8686 |
| | SUIT2 |
| | TCCPAN2 |
| | YAPC |

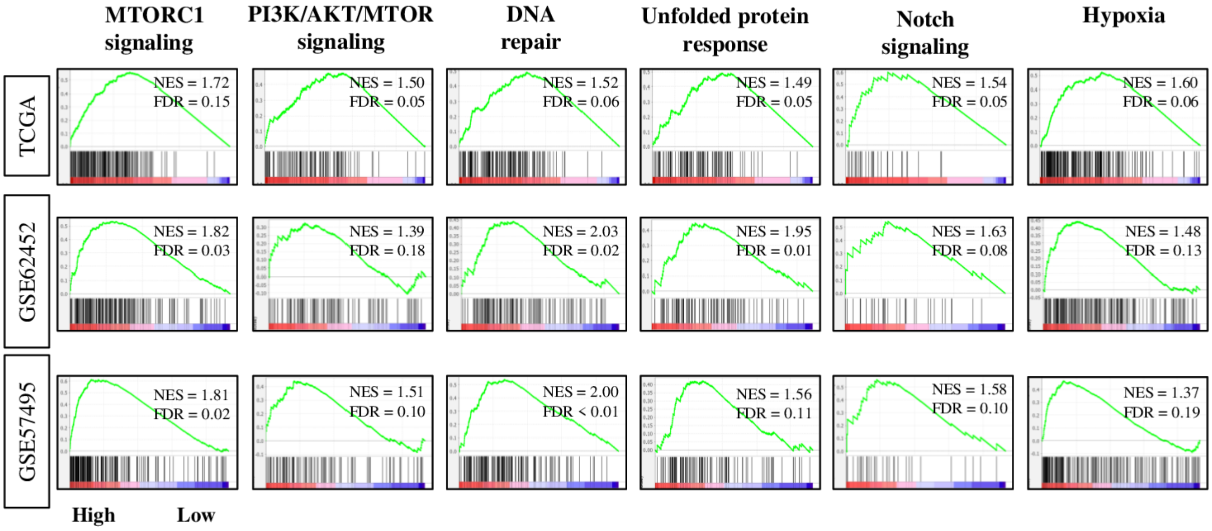


**Figure S1.** Gene set enrichment analysis (GSEA) of high 4-gene score pancreatic cancer in the TCGA, GSE62452, and GSE57495 cohorts. Enrichment plots along with normalized enrichment score (NES) and false discovery rate (FDR) are shown for cancer aggressiveness-related Hallmark gene sets, including MTORC1 signaling, PI3K/AKT/MTOR signaling, DNA repair, unfolded protein response, notch signaling, and hypoxia. NES and FDR were determined with the classical GSEA method, where FDR < 0.25 is considered significant.
